# Scale‐Up of Innovative Honeycomb Reactors for Power‐to‐Gas Applications – The Project Store&Go

**DOI:** 10.1002/cite.201700139

**Published:** 2018-04-14

**Authors:** Dominik Schollenberger, Siegfried Bajohr, Manuel Gruber, Rainer Reimert, Thomas Kolb

**Affiliations:** ^1^ Karlsruher Institute of Technology Engler-Bunte-Institut Department of Fuel Technology Engler-Bunte-Ring 1 76131 Karlsruhe Germany

**Keywords:** Catalyst carriers, Energiewende, Energy storage, Formal kinetics, Methanation, Power‐to‐X

## Abstract

The German “Energiewende” is heavily based on electric power and, therefore, requests solutions to serve non‐electric energy uses and to store electric energy in large scale. Synthetic natural gas (SNG) produced with hydrogen from water electrolysis and with CO_2_ from mainly renewable sources is one approach. For the catalytic SNG production efficient removal and utilization of the reaction heat is the main issue. A metallic honeycomb‐like carrier‐based reactor proved in laboratory scale to match this challenge. This type of reactor shows good heat conductivity and enables optimized operation. In the EU‐funded project Store&Go the honeycomb methanation is scaled up to MW‐scale. For this, heat transfer and kinetic data were determined experimentally and used in CFD calculations for the reactor design. Finally a SNG plant with 1 MW feed‐in will be built and fully integrated operation will be shown.

## 1 Motivation

**Figure 1:**
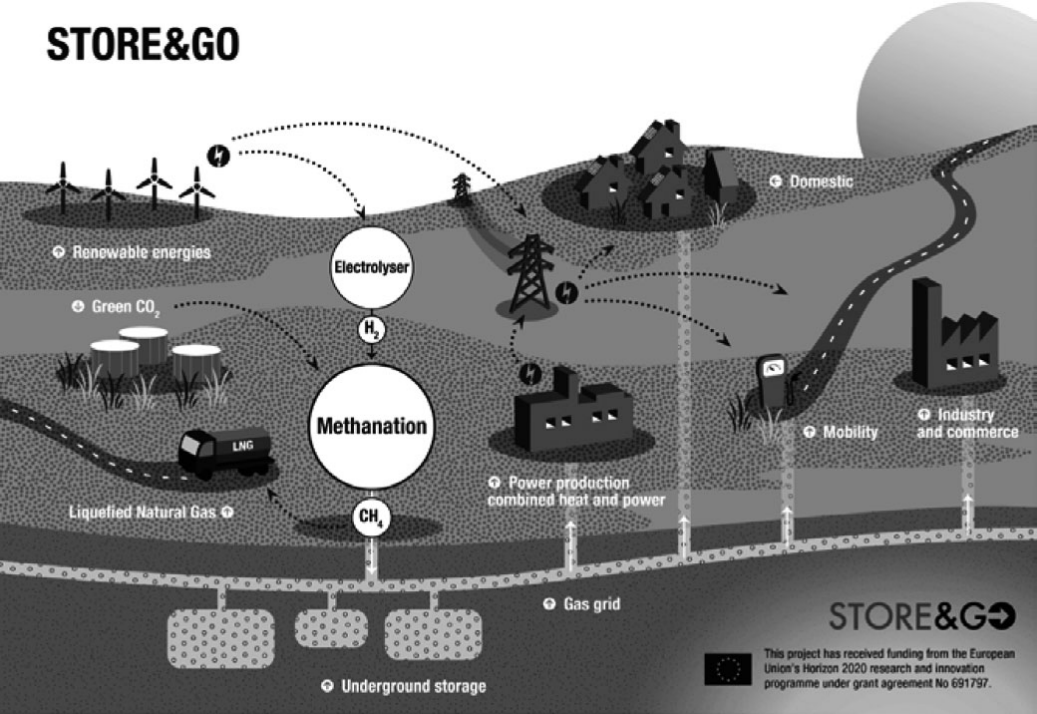
The Store&Go process.

The German Energiewende aims for the nationwide transition of the energy system from fossil to renewable energy supply. Wind and solar power are the dominant renewable sources wherefore solutions to serve non‐electric energy uses and to store electric energy in large scale are required. SNG produced catalytically with hydrogen from water electrolysis and with CO_2_ from various sources but mainly from renewables can be one solution. From Fig. 1 showing the power‐to‐gas (PtG) process schematically it can be seen that it

- enables the coupling of the energy sectors electricity, heat and mobility, and
- increases the flexibility of the energy system.
- The advantages of SNG produced via PtG are 1, 2:
- it is a CO_2_‐neutral fuel with high energy density,
- its properties (e.g., composition, heating value) are comparable to those of natural gas,
- it can therefore be transported, stored and used within the existing gas infrastructure,
- it allows for large‐scale and cost‐efficient energy storage, and
- it has the highest conversion efficiencies of all PtX processes (except direct H_2_ utilization).

## 2 The Project Store&Go

To improve the performance of the catalytic methanation a new type of reactor, the honeycomb methanation reactor, was developed and tested in lab‐scale. In a next step this reactor concept will be demonstrated in a semi‐commercial scale (1 MW equivalent hydrogen flow) at the demonstration site Falkenhagen whose key characteristics are:

- wind power as a renewable energy source,
- 2 MW alkaline electrolyzer,
- integration in a transport grid, and
- excess heat integration in neighboring industries.
- The goals of the demonstration project Store&Go are:
- total operation time of more than 24 months (4000 h),
- operation in the environment of an existing energy grid,
- demonstration of broad part‐load operation (load range: 20 – 100 %), and
- conformity of gas quality with specifications (*y*_CH4_ > 95 vol %).

## 3 Process and Reactor Basics

Since according to Eq. (1) methanation is an exothermic reaction, an efficient removal of the reaction heat is necessary for controlling the reactor 3.

(1) $$\begin{array}{cc} CO_{2}+4H_{2}⇌CH_{4}+2H_{2}O_{\left(g\right)} & \Delta_{R}H^{0}=-165\;\mathrm{kJ}\;\mathrm{mo}l^{-1} \end{array}$$

A reactor consisting of a tube filled with a metallic honeycomb‐like body as carrier system for the methanation catalyst proved to fulfill this requirement effectively. The honeycomb‐like body is made up from a combination of corrugated and plane metal sheets which are jointly coiled up. Due to its structure it offers higher radial heat conductivity than fixed‐bed reactors frequently used in multitube arrangement for exothermic catalytic reactions. Depending on its internal dimensions, the metallic honeycomb body has a high volume‐specific surface area necessary and suitable for efficient catalyst impregnation 4.

The process was developed in lab‐scale and its technical feasibility was proven in a bench‐scale plant (100 kW equivalent hydrogen flow) added to a biomass gasification plant in Köping, Sweden. For designing the 1‐MW plant the whole system consisting of honeycomb, washcoat, reactor tube, and the heat removal system needs to be described. For this purpose experimental investigations were carried out on radial heat transport and on reaction kinetics. For evaluating the experimental results as well as for the dimensioning of the honeycomb reactor the system was mathematically modeled.

### 3.1 Description of Heat Transfer

The effective radial thermal conductivity *λ*_r,eff_ of a honeycomb body depends strongly on the dimensions (wall thickness, porosity) of the internal structure and on the thermal conductivity of the material of construction, but also on the properties of the gas flowing through the structure and its flow velocity. For calculating effective conductivities several correlations were published in literature 5, 6, 7, 8, 9. The correlations were developed from models for monolithic honeycomb structures in which the internal structure is counted for by heat resistors which are interconnected in various ways. For a symmetric interconnection of the resistances Visconti 8 developed Eq. (2) and Eq. (4) for calculating both the radial and the axial thermal conductivity. Eq. (3) is based on Visconti's Eq. (2) excluding the thermal conductivity of the gas phase.

(2) $$\lambda_{r,\mathrm{eff}}=\lambda_{s}\frac{\frac{\lambda_{g}^{2}}{\lambda_{s}^{2}}\frac{\left(1+\varepsilon \right)}{\left(1+\varepsilon \right)}+\frac{\lambda_{g}}{\lambda_{s}}\frac{\left(3\varepsilon^{2}+2\varepsilon +3\right)}{{\left(1+\varepsilon \right)}^{2}}+2\frac{\left(1-\varepsilon \right)}{\left(1+\varepsilon \right)}}{\frac{\lambda_{g}^{2}}{\lambda_{s}^{2}}{\left(\frac{1-\varepsilon }{1+\varepsilon }\right)}^{2}+3\frac{\lambda_{g}}{\lambda_{s}}\frac{\left(1-\varepsilon \right)}{\left(1+\varepsilon \right)}+2}$$

(3) $$\lambda_{r,\mathrm{eff}}=\lambda_{s}\frac{\left(1-\varepsilon \right)}{\left(1+\varepsilon \right)}$$

(4) $$\lambda_{z,\mathrm{eff}}=\lambda_{s}\left(1-\varepsilon \right)+\lambda_{g}\varepsilon$$

Since the internal structures of the monolithic honeycombs modeled so far in literature differ from the structure of the metallic honeycomb developed here an attempt was made to determine experimentally its effective radial thermal conductivity. With the same equipment, the heat transport parameters of monolithic honeycomb bodies were also determined to compare them with the literature.

### 3.2 Experimental Setup for Heat Transport Measurements

**Figure 2:**
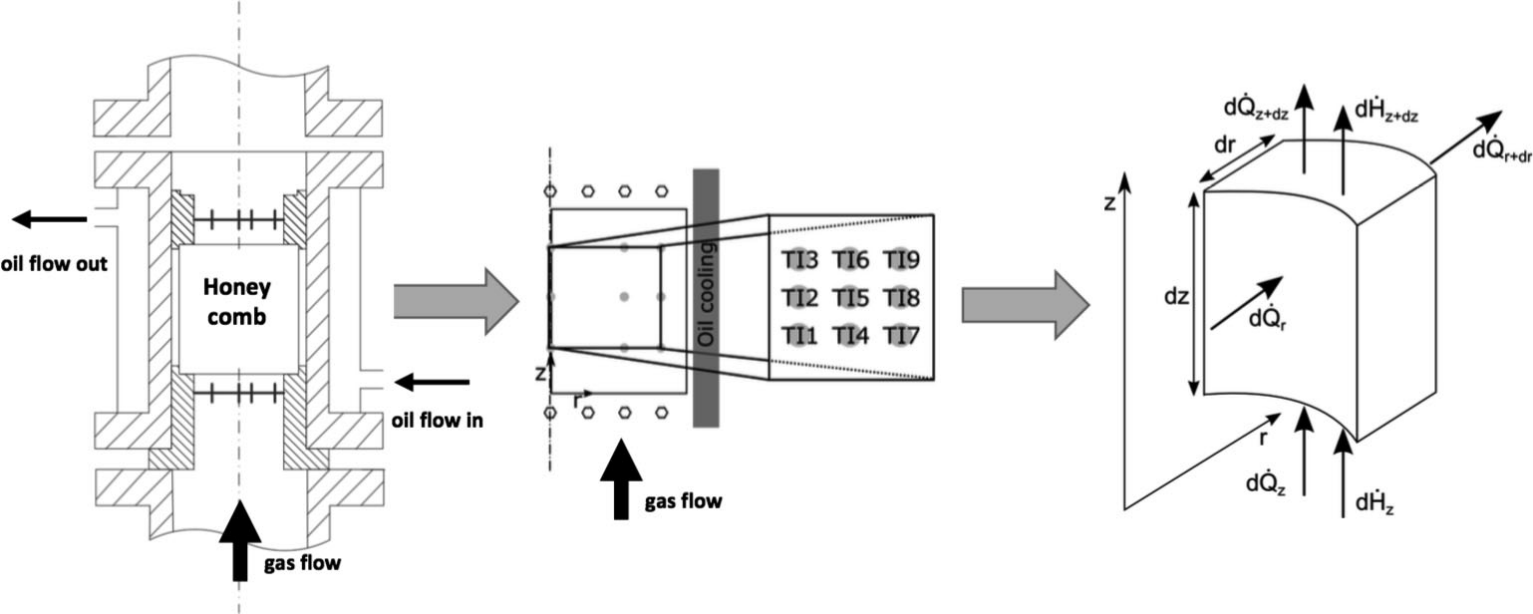
Experimental setup: honeycomb placed in the measuring section (left), the positioning of the thermocouples (middle), and a schematic volume element for balancing (right).

Fig. 2 shows the setup and the data evaluation procedure for measuring temperature profiles in honeycombs with gas flow (without reaction) in order to determine *λ*_r,eff_. The measurements are carried out in a wide range of parameters. The experimental setup consists of a double‐tube measuring section in which the honeycomb bodies with a diameter of 35 mm and a length up to 150 mm are placed. Between the honeycomb bodies and the wall of the inner reactor tube is a gap of less than 0.2 mm. The outer tube is surrounded by a jacket providing heating or cooling via a heat transfer oil. A high oil flow provides for a constant oil temperature over the whole measuring section. Dry and preheated air (100 °C to 400 °C) is routed through the honeycomb bodies during the measurements. A radial temperature gradient can be applied from inside to outside and vice versa depending on the temperature of the oil, which can be varied between 50 °C and 350 °C. The gas temperatures are measured 10 mm upstream and downstream the honeycombs, respectively, at four radial positions, and the honeycombs are equipped with at least nine thermocouples to measure the surface temperatures. The positions of the thermocouples are indicated in Fig. 2. The gas velocity *u*_0,NTP_ was varied between 0.8 and 3 m s^−1^. The pressure *p* was kept at ambient pressure.

The evaluation of the experimental results is based on the energy balance around the honeycomb represented by Eq. (5). The differential equation is numerically solved using a routine in Matlab®. All the properties of the convective energy flow are known. The effective axial heat conductivity *λ*_z,eff_ is calculated using Eq. (4). Thus, the effective radial heat conductivity *λ*_r,eff_ is the only unknown. The temperature fields measured during the experiments are compared to the numerically calculated temperature fields while adapting *λ*_r,eff_. The best adaptation is achieved if the difference between the two temperature fields becomes minimal.

(5) $$\rho_{g}\;c_{p,g}\;u_{0,g\;}\frac{\partial T}{\partial z}=\lambda_{r,\mathrm{eff}}\left(\frac{\partial^{2}T}{\partial r^{2}}+\frac{1}{r}\frac{\partial T}{\partial r}\right)+\lambda_{z,\mathrm{eff}}\frac{\partial^{2}T}{\partial z^{2}}$$

To solve the differential equation, the following boundary conditions were set:

- *T* = *T*_in_(*r*) at *z* = 0 (Dirichlet boundary condition (D‐BC)),
- $\frac{\partial T}{\partial z}=0$ at *z* = *L* (Neumann boundary condition (N‐BC)),
- $\frac{\partial T}{\partial r}=0$ at *r* = 0 (Neumann boundary condition (N‐BC)),
- *T* = *T*_Wall_(*z*) at *r* = *R* (Dirichlet boundary condition (D‐BC)).

**Figure 3:**
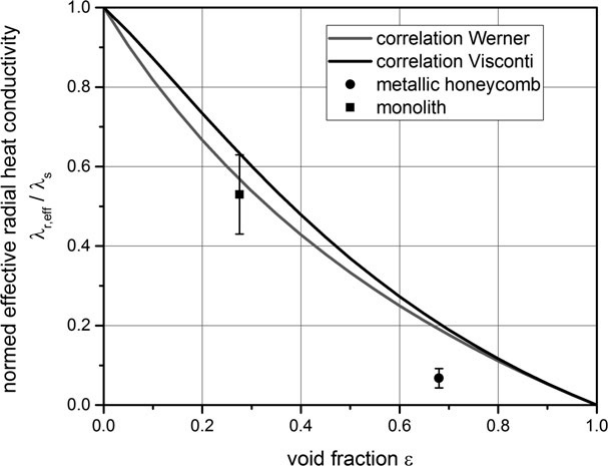
Comparison of experimental results for monoliths and metallic honeycombs with correlations 8, 9. Werner's correlation (Eq. (3)) is based on Visconti's (Eq. (2)) excluding the thermal conductivity of the gas phase.

The experimental results in Fig. 3 show that the values determined for monoliths correspond to those predicted from the correlation. In so far, the applied method, i.e., the experimental setup and the analysis of the results, is validated for use with the metallic honeycomb. Obviously, the correlation is not applicable for the metallic honeycomb.

### 3.3 Investigation of Reaction Kinetics

Ni/Al_2_O_3_ catalysts are often used for the methanation of carbon dioxide. Reaction rate equations can be deduced from surface reaction mechanisms or represented globally (formal‐kinetic) 10, 11, 12. In the simulations for the scale‐up a formal‐kinetic approach is used for reason of better handling in the COMSOL reactor model. For determining the kinetic parameters experiments were carried out in an isothermal fixed‐bed reactor (PFR). The reactor tube has an inner diameter of 15 mm and a length of 500 mm. A catalytic bed of 150 mm length was placed inside, 200 mm downstream the gas inlet of the reactor. The catalyst particle sizes range from 180 to 250 µm. The bed was diluted with silicon carbide with the same particle fraction in a ratio of 1/20 resulting in a catalyst mass of 2 g. The gas is preheated when passing an inert bed of 50 mm length, before entering the catalytic bed. The reaction parameters were varied within a wide range ($p_{CO_{2}}$ = 0.1 – 4 bar, *T* = 200 – 300 °C, *p*_abs_ = 2 – 17 bar) resulting in Eq. (6) for the reaction rate and Eq. (7) for the rate coefficient. As at all measured points the Mears‐, Anderson‐ and Weisz‐Prater criterion was fulfilled neither intraparticle nor external mass and heat transport limitations were expected.

(6) $$r_{m,CH_{4}}=k_{\mathrm{Metha}}p_{CO_{2}}^{0.47}p_{H_{2}}^{0.54}\left(1-\frac{Q_{\mathrm{Metha}}}{K_{p,\mathrm{Metha}}}\right)$$

(7) $$k_{\mathrm{Metha}}=9.98\cdot 10^{5}\exp\left(-\frac{84\;\mathrm{kJ}\;\mathrm{mo}l^{-1}}{\mathrm{RT}}\right)\frac{\mathrm{mol}}{\mathrm{kg}\;s\;\mathrm{ba}r^{1.01}}$$

**Figure 4:**
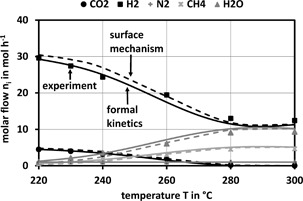
Comparison of results from formal kinetics (Eq. (6)) and the surface mechanism with experimental results for the methanation of CO_2_ with a stoichiometric feed.

The activation energy *E*_A_ of 84 kJ mol^−1^ is quite low but in the range published in literature. Parity plots for the experimental results and Eq. (6) show that 95 % of the values are within a spread of ± 10 %. The applicability of the results gained in an isothermal fixed‐bed reactor for the non‐isothermal reaction in channels was proven experimentally. These experiments were carried out both in an isothermal and in a non‐isothermal honeycomb reactor and the results will be published later. Fig. 4 compares the power‐law kinetics with experiments from the isothermal fixed‐bed reactor and the surface reaction mechanism.

## 4 Scale‐Up

**Figure 5:**
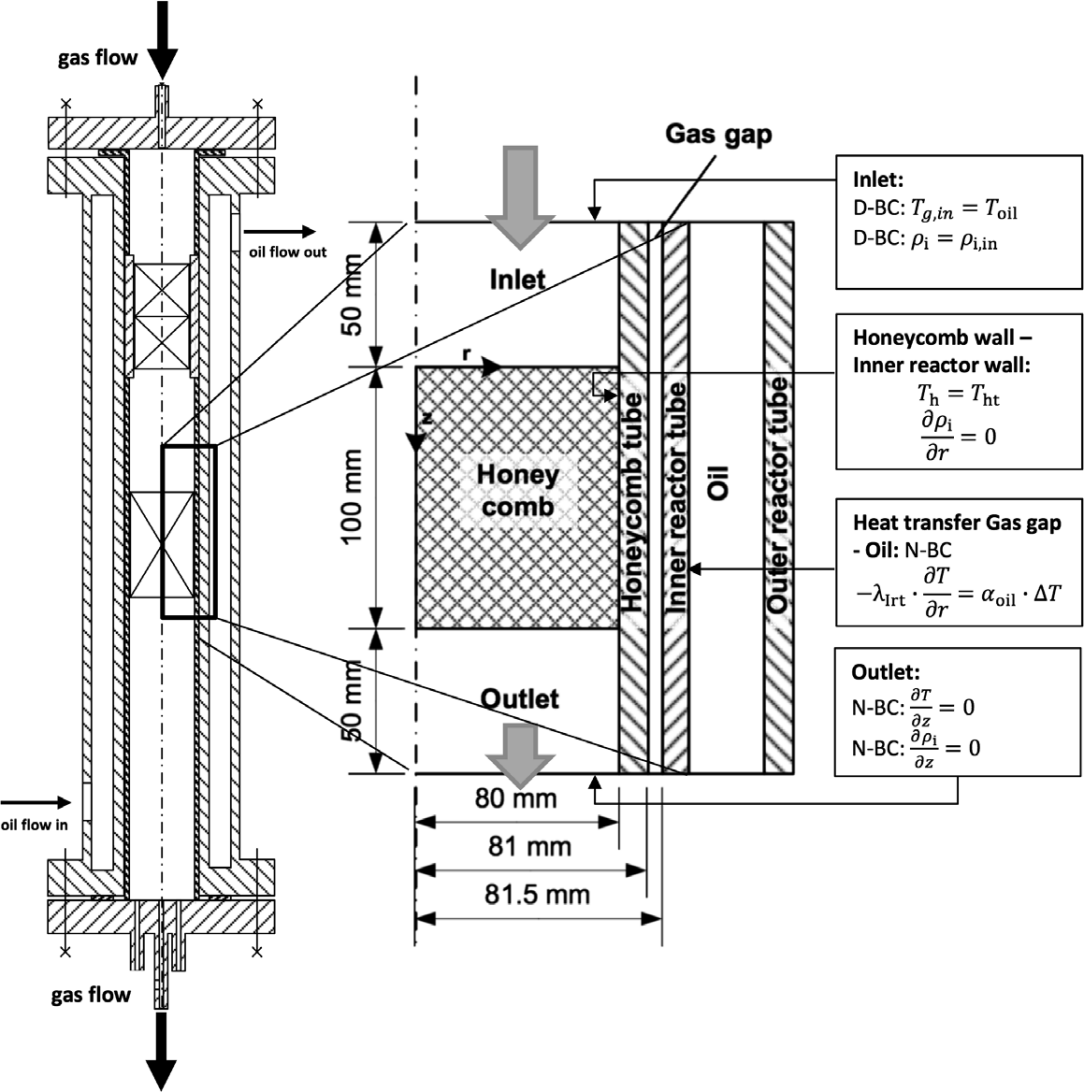
Reactor geometry and boundary conditions for the COMSOL model.

For pure fluid processes the scale‐up from lab to demo‐scale can be made rather reliably by numerical modeling. For this purpose, a continuous, quasi‐homogeneous, stationary, two‐dimensional, rotational symmetric COMSOL model was developed based on the detailed geometry of one single tube of the multitube honeycomb reactor. Fig. 5 shows the implemented geometry and the boundary conditions of the COMSOL model. The power‐law kinetics (Eq. (6)) and the measured *λ*_r,eff_ were implemented. The goal of the design by scale‐up is to find an optimized reaction path which means a high reaction rate with small reactor dimensions. From the exothermic nature of the methanation reaction the need for a high radial heat flux infers. With a multi‐parameter optimization parameter sets can be determined to reach defined temperature profiles in the reactor tubes. The COMSOL optimization module served for parameter optimization was used. The model was experimentally validated with experimental setups with honeycomb diameters from 35 mm up to 105 mm and lengths from 100 mm up to 500 mm.

The produced SNG shall be injected into the gas grid. In order to fulfill the applicable regulations for gas injection into the gas grid in Germany a methane content of more than 95 vol % in the final SNG is mandatory 11. This methane content is achieved at a CO_2_ conversion rate of at least 99 %. However, the thermodynamic equilibrium sets the limits for the achievable conversion rates, so that the temperature at the outlet has to be below 260 °C. At this temperature level the reaction rate is almost prohibitively low. With respect to the economy, the flexibility of the load and the integration of small plants into the existing infrastructure, a process concept is required which needs as few reactor steps as possible. In the case of the honeycomb reactors the required conversion can be reached with only two reaction zones. A kinetically controlled zone at the inlet with the maximum possible reaction rate at high temperatures is followed by a thermodynamically controlled reaction zone which adjusts the necessary equilibrium composition at the outlet at low temperatures.

**Figure 6:**
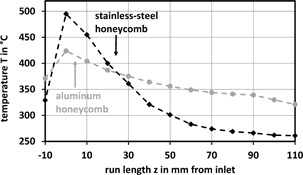
Axial temperature profiles at *r* = 0 for a stainless‐steel honeycomb and an aluminum honeycomb with an oil temperature of *T* = 260 °C and *u*_0,NTP_ = 0.1 m s^−1^.

**Figure 7:**
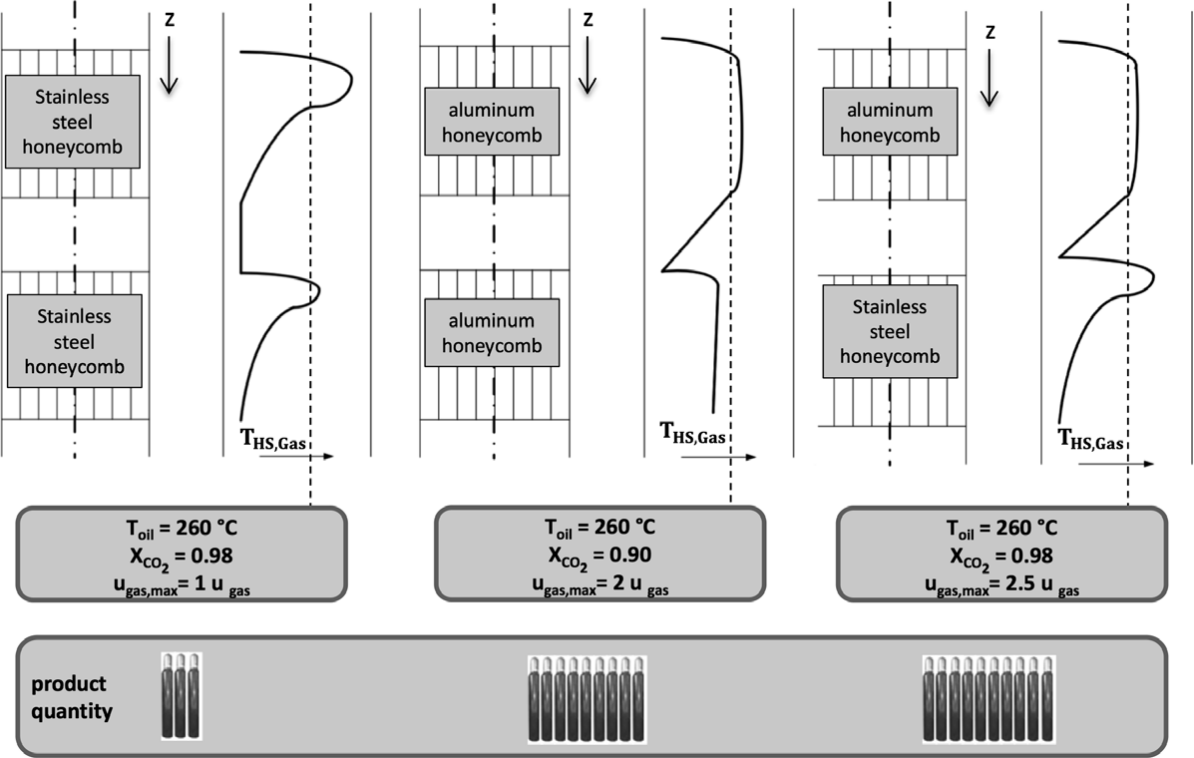
Schematic combinations of aluminum and stainless‐steel honeycombs with trends for the maximum allowable gas velocity and the corresponding conversion rate of CO_2_. The gas bottles represent a relative gas production rate.

Fig. 6 shows typical experimental temperature profiles at the first centimeters for a stainless steel and for an aluminum honeycomb, respectively. For the stainless steel honeycomb, the temperature has a pronounced peak at the inlet and goes down close to the oil temperature at the outlet. In contrary, the aluminum honeycomb has no such pronounced temperature peak because of its 20 times higher heat conductivity *λ*_s_, but a higher temperature at the outlet 12. The higher outlet temperature restricts the CO_2_ conversion due to the thermodynamic equilibrium where the gas composition does not meet the specifications. However, due to the integrally higher temperature level in an aluminum honeycomb a larger specific product quantity can be reached than in a steel honeycomb (Fig. 7). The experimental conditions are close to the technical reactor, the gas velocity *u*_0,NTP_ can be in the range from 0.1 to 1 m s^−1^ and the oil temperature *T* from 200 to 320 °C.

From the results, a combination of an aluminum and a steel honeycomb looks promising. Fig. 7 shows the production figures for two honeycombs equal in size but different in materials and for the combination of an aluminum honeycomb in front followed by a stainless‐steel honeycomb. The combination benefits from the high specific production rate of the aluminum part and from the low radial conductivity of the steel part, which avoids fast cooling of the gas and, hence, allows for the high CO_2_ conversion.

As in the case of the multitube fixed‐bed reactors, the scale‐up of a honeycomb reactor means to find the greatest allowable honeycomb diameter. The honeycomb diameter is dominantly limited by the radial heat conductivity of the matrix. Based on experimental results and results from the multi‐parameter optimization a diameter of approx. 80 mm is an optimum for this application.

## Symbols used

*c*_p_ [J kg^−1^K^−1^]: specific heat capacity at constant pressure
*E*_A_ [kJ mol^−1^]: activation energy
*H* [J mol^−1^]: molar enthalpy
Δ_R_*H*^0^ [J mol^−1^]: molar standard reaction enthalpy
*k*_Metha_ [mol kg^−1^s^−1^$\mathrm{ba}r^{-z_{\mathrm{ges}}}$]: catalyst mass related reaction coefficient
*K*_p_ [$\mathrm{ba}r^{z_{\mathrm{ges}}}$]: equilibrium coefficient
*p* [Pa]: pressure
*p*_i_ [Pa]: partial pressure
*m* [kg]: mass
*n*_i_ [–]: molar flow of *i*
$\overset{\cdot }{Q}$ [J s^−1^]: heat flow
*Q*_p_ [–]: term for the current composition of the reaction mixture
*r* [m]: radius
*r*_m,i_ [mol kg^−1^s^−1^]: catalyst mass related formation rate of *i*
*R* [J K^−1^mol^−1^]: universal gas constant
*T* [K, °C]: temperature
*u*_0,NTP_ [m s^−1^]: superficial velocity at standard conditions
*X*_i_ [–]: conversion rate of *i*
*z* [m]: run length
*z*_ges_ [–]: overall reaction order

**Greek symbols**
*ε* [–]: porosity
*λ* [W m^−1^K^−1^]: heat conductivity
*λ*_r,eff_ [W m^−1^K^−1^]: effective radial heat conductivity
*λ*_z,eff_ [W m^−1^K^−1^]: effective axial heat conductivity
*ρ*_g_ [kg m^−3^]: gas density

**Sub‐ and Superscripts**
eff: effective
g: gas
h: honeycomb
ht: honeycomb tube
i: species
in: inlet
Irt: inner reactor tube
max: maximum
oil: oil
r: radial
s: solid
z: axial

**Abbreviations**
D‐BC: Dirichlet boundary condition
N‐BC: Neumann boundary condition
NTP: standard conditions (0 °C and 1013 mbar)
PtG: Power to gas
PtX: Power to X
SNG: synthetic natural gas

## References

[1] M. Götz , J. Lefebvre , F. Mörs , A. McDaniel Koch , F. Graf , S. Bajohr , R. Reimert , T. Kolb , Renewable Energy 2016, 85, 1371 – 1390. DOI: 10.1016/j.renene.2015.07.066

[2] M. Bailera , P. Lisbona , L. M. Romeo , S. Espatolero , Renewable Sustainable Energy Rev. 2017, 69, 292 – 312. DOI: 10.1016/j.rser.2016.11.130

[3] S. Rönsch , J. Schneider , S. Matthischke , M. Schlüter , M. Götz , J. Lefebvre , P. Prabhakaran , S. Bajohr , Fuel 2016, 166, 276 – 296. DOI: 10.1016/j.fuel.2015.10.111

[4] E. Tronconi , G. Groppi , T. Boger , A. Heibel , Chem. Eng. Sci. 2004, 59 (22 – 23), 4941 – 4949. DOI: 10.1016/j.ces.2004.07.018

[5] G. Groppi , E. Tronconi , AIChE J. 1996, 42 (8), 2382 – 2387. DOI: 10.1002/aic.690420829

[6] R. E. Hayes , A. Rojas , J. Mmbaga , Catal. Today 2009, 147, S113 – S119. DOI: 10.1016/j.cattod.2009.07.005

[7] A. G. Konstandopoulos , M. Kostoglou , N. Vlachos , E. Kladopoulou , Adv. Chem. Eng. 2007, 33, 213 – 294. DOI: 10.1016/S0065-2377(07)33004-4

[8] C. G. Visconti , G. Groppi , E. Tronconi , Chem. Eng. J. 2013, 223, 224 – 230. DOI: 10.1016/j.cej.2013.02.095

[9] K. Werner , Fortschr.‐Ber. VDI 1993, 3 (306), 1 – 220.

[10] W. Wei , G. Jinlong , Front. Chem. Sci. Eng. 2011, 5 (1), 2 – 10. DOI: 10.1007/s11705-010-0528-3

[11] Technische Regel – Arbeitsblatt G. 262 (A), *Nutzung von Gasen aus regenerativen Quellen in der öffentlichen Gasversorgung*, Wirtschafts‐ und Verlagsgesellschaft Gas und Wasser, Bonn 2011.

[12] VDI‐Wärmeatlas, 11th ed., Springer Vieweg, Heidelberg 2013.

